# Quality by Design Approach for the Formulation and Evaluation of Stem Cells Derived Rosmarinic Acid-Loaded Nanofibers as an Anti-Wrinkle Patch: In Vitro and In Vivo Characterizations

**DOI:** 10.3390/pharmaceutics16121598

**Published:** 2024-12-16

**Authors:** Rehab Abdelmonem, Ahmed Bakr, Ingy Badawy, Ahmed Ibrahim Abd El Maksoud, Reem T. Attia

**Affiliations:** 1 Industrial Pharmacy Department, College of Pharmaceutical Sciences and Drug Manufacturing, Misr University for Science and Technology (MUST), 6th of October City, Giza 12566, Egypt; rehab.abdelmonem@must.edu.eg (R.A.); ahmed.bakr@must.edu.eg (A.B.); 2 Pharmaceutical and Industrial Biotechnology Department, College of Biotechnology, Misr University for Science and Technology (MUST), 6th of October City, Giza 12566, Egypt; inji.mahmoud@must.edu.eg; 3 Industrial Biotechnology Department, Genetic Engineering and Biotechnology Research Institute, University of Sadat City, Sadat City 32897, Egypt; ahmed.ibrahim@gebri.usc.edu.eg; 4 Department of Pharmacology, and Toxicology, and Biochemistry, Faculty of Pharmacy, Future University in Egypt, Cairo 11865, Egypt

**Keywords:** anti-wrinkle, plant stem cells, *Salvia miltiorrhiza*, rosmarinic acid, electrospun nanofiber, Nrf2/Keap1

## Abstract

**Background/Objectives:** Skin wrinkles result from a myriad of multifaceted processes involving intrinsic and extrinsic aging. To combat this effect, plant stem cells offer a renewable and eco-friendly source for various industries, including cosmeceuticals. *Salvia miltiorrhiza* (SM), which contains the bioactive compound Rosmarinic acid (RA) and has been proposed for its anti-wrinkle effect. **Methods:** In the present study, calli from SM were cultured and Quality by Design (QbD) was implemented to investigate the effect of different types and concentrations of elicitors; jasmonic acid (JA) and salicylic acid (SA). Both raised RA levels yet, jasmonic acid (50 µM) has resulted in the highest yield for RA, at 16 mg/g. A nanofiber patch was prepared and characterized in-vitro by the release percentage, drug content, swelling degree, scanning electron microscope, and surface roughness. Then, the anti-wrinkle effect of the patch was tested in a UV wrinkle-induced mouse model. **Results:** Interestingly, after treatment, there were visibly fewer wrinkles, and the skin was softer than in the untreated control group. This study suggests that the treatment exerted its effect through the Nrf2/Keap1 pathway, which plays a crucial role in cellular antioxidant protective processes. By activating this pathway through boosting Nrf2 and diminishing Keap1 cellular content, the nanofiber patch enhances the production of antioxidant enzymes, such as superoxide dismutase and glutathione peroxidase, enhancesglutathione, and reduces the skin lipid peroxidation, collectively indicating enhanced skin quality. **Conclusions:** In conclusion, this study highlights the importance of this formula as an anti-wrinkle treatment, and future clinical studies are recommended to further unveil the potential of this formula.

## 1. Introduction

Wrinkles are visible creases or folds in the skin [1]. Skin wrinkles cause gradual changes in the structure and function of the skin [2]. Wrinkle formation is a result of intrinsic or extrinsic factors [3]. Intrinsic aging is a natural physiological process characterized by fine wrinkles and dry skin. In contrast, environmental factors, including air pollution and sun exposure, can cause extrinsic aging [4]. Ultraviolet rays (UVRs) are the primary extrinsic skin aging factor responsible for free radical generation [5]. Plant stem cells are fundamentally undifferentiated cells present in the meristematic tissues of plants [6]. Plant stem cell-based cosmeceutical products have various applications, including enhancing the elasticity of the epidermis and serving as anti-wrinkle agents [7].

Various plant extracts have been shown to exert pharmacological effects due the flavonoids, polyphenols, and numerous other bioactive substances found in these extracts, which have important therapeutic applications [8]. One of these notable plants is *Salvia miltiorrhiza (SM)*, which holds a prominent position among China’s most popular traditional herbal medicines [9]. This plant, commonly known as Danshen, has been extensively utilized in traditional Chinese medicine due to its broad spectrum of therapeutic properties [10]. The major phenolic acid compounds present in SM include caffeic acid, Danshensu, Rosmarinic acid (RA), and salviolinic acid [9]. In fact, caffeic acid is well regarded for its antioxidant and anti-inflammatory effects, which contribute to the plant’s overall medicinal efficacy [11]. Danshensu, a unique phenolic compound, demonstrates cardioprotective properties, making it valuable for treating cardiovascular diseases [12]. Furthermore, Rosmarinic acid (RA) is another significant phenolic acid known for its anti-inflammatory and antimicrobial properties and strong antioxidant activity [12], Thus, RA is believed to abate skin aging due to its capability to stabilize free radicals, which accumulate in the skin due to sun exposure and environmental pollution, inducing wrinkle formation [13]. Moreover, salviolinic acid is believed to contribute to the plant’s overall health benefits through its various bioactive effects. Together, these compounds synergistically contribute to the diverse pharmacological activities of SM, strengthening its importance as a component in traditional herbal medicine [11].

Several studies have proven that plant hormones such as Salicylic acid (SA) and Jasmonic acid (JA) may induce the accumulation of important medicinal secondary metabolites like phenolic acids [14,15,16]. By the same token, in this study, SA and JA increased the levels of RA. Nanotechnology in drug delivery has revolutionized the field by enabling precise drug delivery systems, which can be utilized in a wide range of applications, extending from targeted cancer therapy [17] to targeted advanced skincare formulations [18]. Some of these delivery systems are nano-emulsions, solid lipid nanoparticles, nanostructured lipid carriers, liposomes, and niosomes [19]. Nanofibers are the category of nano-formulations that have emerged as a transformative technology in many medical fields such as wound healing and anti-wrinkle formulation; also, the residence time of nanofibers is considered longer time than the mentioned types [20,21]. In anti-wrinkle formulas, nanofibers such as carbon-based, polymeric, and composite nanofibers facilitate the profound penetration of active ingredients into the skin [20]. This targeted delivery system boosts collagen production and skin elasticity, leading to more pronounced and sustained anti-aging effects [19].

Quality by Design (QbD) is a systematic methodology used in manufacturing, biotechnology, and pharmaceutical research and development. It prioritizes a science-based understanding of the product and process. Additionally, QbD encourages optimization and continual improvement throughout the study life cycle by identifying areas for improvement and making iterative adjustments utilizing data-driven decision-making methods [21].

An efficient method for nanofiber preparation is electrospinning; this technique involves the application of a high-voltage current to a polymer solution, which is ejected from a syringe needle to form ultra-thin fibers. The electrostatic forces overcome the surface tension of the polymer solution, stretching it into fine fibers as it travels toward a grounded collector [22]. A polyurethane polymer was used as it has self-healing and anti-aging properties [23].

Oxidative stress plays a crucial role in the formation of wrinkles by damaging cellular components such as lipids, proteins, and DNA [24]. This damage results from an imbalance between the production of reactive oxygen species (ROS) and the skin’s antioxidant defenses. As ROS accumulate, they accelerate the appearance of fine lines and wrinkles [25]. The Nuclear factor erythroid 2-related factor 2 (Nrf2)/Kelch-like ECH-associated protein 1 (Keap1) pathway is known to orchestrate the expression of various antioxidant proteins that help mitigate oxidative damage, a primary driver of skin aging and wrinkle formation [26]. Thus, the current study investigates the mechanistic effect of SM extract through this pathway as an anti-wrinkle formulation. The aim of this study is to prepare a natural dosage form optimized through QbD and in-vivo study as an anti-wrinkle treatment.

## 2. Materials and Methods

### 2.1. Materials

*Salvia miltiorrhiza* came from Zhongjiang (Zhongjiang, China). Rosmarinic acid, Formaldehyde, Xylene 99%, Paraffin, N, N-dimethylformamide (Anhydrous, 99.8%, DMF), and 2,4-dichlorophenoxyacetic acid were purchased from (Sigma-Aldrich, Burlington, MA, USA). Sodium hypochlorite solution was purchased from (Fuchen Tianjin Chemical Reagent Factory, Tianjin, China). Murashige and Skoog (MS) medium was purchased from (Caisson Laboratories, Smithfield, Alexander, AR, USA). Agar was purchased from (Mira Lab in Cairo, Egypt). Salicylic acid was purchased from (El Gomhouria Company, Cairo, Egypt). Jasmonic acid was purchased from (Cayman Chemical Company, Ann Arbor, MI, USA). Methanol (99% pure), acetone (99.7% pure), and Glacial Acetic acid (99.85% pure) were purchased from (Pioneers for Chemicals (PIOCHEM) Company, Giza, Egypt). Anhydrous sodium acetate U.S.P. 37 was purchased from (EL Nasr Pharmaceutical Chemicals Company, Cairo, Egypt). The polymer polyurethane Tecoflex EG-80A was purchased from Noveon, Inc. (Brecksville, OH, USA). ELISA kits (My BioSource, San Diego, CA, USA) and colorimetric kits were from (Biodiagnostic, Giza, Egypt).

### 2.2. Callus Culture from Shoot Tip Meristematic Stem Cells of Salvia miltiorrhiza

Explants of leaves and stems from SM were cultured on the same day as harvest. Afterward, the samples were immersed in distilled water after cleaning with tap water. Subsequently, explants of leaves and stems were surface sterilized for 10 s in 80% ethanol, rinsed twice with sterile distilled water, soaked for 10 s in 20% sodium hypochlorite solution, and rinsed three times with sterile distilled water while operating in a laminar flow. According to a previous protocol by Ching et al., 2016, sections of sterilized shoot tip explants measuring one centimeter in length were grown in (MS) medium [27]. The medium contained 40 g/L sucrose as a carbon source, 8 g/L agar for gelling, and growth regulators, such as 2,4-dichlorophenoxyacetic acid (2,4-D, 1 mg/L). The culture medium was adjusted to pH 5.8 for callus initiation, and the culture jars were maintained in the dark at 24 °C. Later, after 4 weeks, callus induction was observed on the surface of the cut edges. The calli collected from each jar were gently pressed on filter paper to remove excess water, and the fresh weights were recorded. Dry weights were recorded after the calli were dried in an oven at 30 °C for 24 h until the weight remained constant. The dry weight samples were ground into fine powder using liquid nitrogen. All experiments were performed in triplicate [28]. The calli grew after 20 days of culture at 27 ± 0.5 °C in the dark. Five subcultures were performed at 20-day intervals to maintain sufficient stock plants for the experiments [29].

### 2.3. Statistical Optimization for Eliciation Process

To examine the effects of JA and SA on RA level in SM callus culture, the experimental data were cross-referenced using D-Optimal Design of the Design-Expert software version 13 (Design-Expert^®^ software, Stat-Ease, Minneapolis, MI, USA), as shown in Table 1 [30].

### 2.4. Eliciation Treatment and Effect of Different Concentrations of Jasmonic and Salicylic Acid Elicitors on Rosmarinic Acid RA Concentration

Three different concentrations of JA (50 µM, 100 µM, 150 µM) and SA (1 µM, 1.5 µM, 2 µM, 4 µM, 8 µM) were applied to sub-cultured calli. The experiment was performed in triplicate. A set of cultures without elicitors served as the controls. The cultured jars were incubated in a growth room at 25 ± 2 °C in darkness to encourage callus formation and then subjected to statistical analysis using Design Expert software to determine the best elicitor for increasing RA levels in SM callus culture as previously described [31].

### 2.5. Extraction of Rosmarinic Acid and Determination of Its Level in Salvia miltiorrhiza Callus

The sub-cultured cells were harvested 3 weeks after the eliciation, even the control group that did not undergo eliciation. Afterward, a freeze-drying process was applied to all the plant cells, which were subsequently ground into powder. Three repetitions of each treatment were conducted as mentioned [32]. 

#### 2.5.1. Ultrasonic-Assisted Extraction of Rosmarinic Acid RA

Ten milliliters of 75% aqueous methanol were used to extract the 0.1 g sample powder using sonication via (Elmasonic, S60, Singen am Hohentwiel, Germany) twice at room temperature for 30 min at 4000 Hz. The supernatants were collected by centrifugation via (SIGMA, 3-30KHS refrigerated centrifuge, Osterode am Harz, Germany) for 15 min at 14,000 rpm and 4 °C during chilling as mentioned previously [33].

#### 2.5.2. Determination of Rosmarinic Acid RA Levels Using UV Spectrophotometer

The supernatant collected from centrifugation was passed through a 0.22-micrometer filter before analysis using a UV spectrophotometer [32]. The UV spectrum of the extracts in methanol showed a shoulder at 290 nm and maximum absorbance at 328 nm, which agrees with the RA standard [33].

### 2.6. Electrospinning Nanofiber Formulation of Extracted Rosmarinic Acid

To create an ideal electrospinning solution and prepare the nanofiber patch, polyurethane (PU) was dissolved in dimethyl formamide–acetone (1:1), which had a 10% weight percentage. The mixture was agitated for 24 h at 50 °C before sonication to prevent aggregation. A blunt-end stainless steel needle was used to fill the syringe with a predetermined amount of PU solution (5 mL). Aluminum foil was placed on top of the grounded steel collector (area: 20 cm^2^) as the backing layer. The electrospinning was performed under ideal conditions through an electrospinning device (SISTANA ES LAB, SBS, Tehran, Iran); the needle tip and grounded collector were spaced 10 cm apart and a voltage of 25 kV was used to create a uniform nanofiber at a flow rate of 0.8 mL/h and a needle gauge of 23 for 7.5 h. Using coaxial electrospinning, the extract containing RA with a concentration of 1 mg/mL was electrospun with polyurethane polymer as mentioned previously [34].

### 2.7. In-Vitro Characterization of Extracted Rosmarinic Acid Electrospun Nanofiber Patch

RA electrospun nanofiber was characterized in vitro through the following methods:

#### 2.7.1. Drug Content Percentage

To determine the drug content (DC%) of the RA electrospun nanofiber patch, 1 mg of RA was dissolved in 20 mL 75% methanol and sonicated at 4000 Hz for 20 min at 37 °C. The drawn sample was passed through a 0.22-micrometer filter, then the analysis was performed using a UV spectrophotometer 328 nm (Biochrome, Libra S60 PC Double Beam Spectrophotometer, Cambridge, UK). The following equation was used to calculate the (DC%) [35]:

DC% = (Actual drug content)/(Theoretical Drug content) × 100(1)

Equation (1): Drug content percentage (DC) Drug Content.

#### 2.7.2. In Vitro Release of Rosmarinic Acid-Loaded Nanofiber Patch

A 2 cm × 2 cm section containing 1 mg of extracted Rosmarinic acid was dissolved in 50 mL of sodium acetate buffer solution (pH 5.5) to mimic the skin pH [36] and was added to Hanson dissolving equipment (Hanson, Vision^®^ G2 Elite 8™, Chatsworth, CA, USA) to evaluate the release of RA from the nanofibrous patch. The dissolution vessels were maintained at 37 ± 0.5 °C. To maintain a constant volume, 3 mL samples were removed from the release media at predetermined intervals (up to 3 h) and replaced with fresh media of equal volume. The release studies were performed in triplicates. The mean cumulative drug release percentage was plotted against time. The amount of drug released from the fibers was determined by converting the absorbance of RA in the buffer solution, which was read using a Libra Biochrome spectrophotometer at a wavelength of 328 nm; the RA concentration was determined according to the calibration curve of RA in the same buffer [37].

#### 2.7.3. Swelling Degree of Extracted Rosmarinic Acid Electrospun Nanofiber Patch

The weights of the patch before and after soaking in the acetate buffer (pH 5.5) for 24 h were compared to determine the extent of swelling. The following equation was used to calculate the degree of swelling [38]:

S% = (Ws−Wd)/Wd × 100(2)

Equation (2) is used to calculate the swelling degree, where

S% = swelling degree (%) (g/g);

Ws = swollen weight (g);

Wd = dry weight (g).

#### 2.7.4. Mechanical Strength of the Rosmarinic Acid-Loaded Nanofiber Patch

Mechanical strength (tensile strength) for the formula was tested through The Mecmesin Multi-Test 2.5-i tensile testing machine (2.5-i, Mecmesin Ltd., Slinfold, UK), which provides precise control over the applied force and displacement. The fibers were carefully mounted and aluminum foil grips were used to minimize slippage during testing; the following equation was used to calculate the tensile strength for the prepared patch [39]:

(3) $$\sigma =\rho_{m}\frac{F}{m}L$$

Equation (3) is used to calculate the tensile Strength, where M is the mass of the sample (measured in mg), ρm is the material density, F is the force (measured in N), L is the initial length of the sample (measured in mm), and σ is the stress expressed in MPa.

#### 2.7.5. Fourier Transform Infrared Spectroscopy (FTIR) Analysis

The suggested structure of RA was confirmed using a Nicolet iS 50 Fourier Transform Infrared Spectrometer (ThermoScientific, Waltham, MA, USA). The material was mixed with KBr to create a disk, which was scanned between 4000 and 400 cm^−1^ at a resolution of 4 cm^−1^. By the same token, the RA nanofiber patch was scanned between 4000 and 400 cm^−1^ at a resolution of 4 cm^−1^ as well [37].

#### 2.7.6. Impact of Storage on the Prepared Electrospun Nanofiber Patch on Drug Content and In Vitro Release

The stability of the patch from the point of view of drug loading content was investigated under various conditions (25 °C ± 60% relative humidity) after three months of patch preparation, and then DC% was calculated as mentioned before.

An in vitro dissolution study was also performed for the prepared patch to ensure its stability over three months; from preparation, 1 mg of each sample was submerged in 50 mL of sodium acetate buffer solution (pH 5.5) and placed in the Hanson dissolution apparatus [40].

#### 2.7.7. Scanning Electron Microscope (SEM) and Surface Roughness

The morphology of the nanofibers patch was examined using an electron microscope (SEM) (EVA MA 10, ZEISS, New York, NY, USA). To begin with, gold particles were applied to the surface of the samples. The average fiber diameter was determined Using software, which is Image J (Image J^®^ software, V1.54, Olympus Analysis, Waltham, MA, USA) [41].

In fact, the roughness of the surface is a defining characteristic of particles. By definition, a surface’s vertical height fluctuations are used to calculate its roughness compared to the surface’s reference mean height [42]. The surface roughness tests were conducted using Surface Metrology Software (https://www.cybertechnologies.com/systems/ct600s/, 8 December 2024 CT600s, Cyber Technologies, Dieters Heim, Germany) [43].

### 2.8. In Vivo Studies

#### 2.8.1. Animals

Thirty adult male Swiss Albino mice weighing 20–30 g were acquired from the National Research Center in Cairo, Egypt and were acclimatized for one week. The use of male mice in the experiment was based on a prevention strategy related to hormonal influences and their potential variability in female skin response. Male mice were chosen to minimize these hormonal fluctuations that are more prominent in females and could introduce additional variability, potentially confounding the results of the anti-wrinkle efficacy evaluation. However, we recognize the importance of including both sexes to ensure comprehensive findings. We will include an explanation of this choice in the manuscript and acknowledge the limitations. Throughout the experiment, they were kept in normal polypropylene cages with four per cage and were subjected to a suitable temperature (25 ± 2 °C), humidity (60–70%), and light (12 h dark/light cycles). Food and water provided for the mice were standard. The U.K. Animals (Scientific Procedures) Act, 1986, and related requirements, such as the EU Directive 2010/63/EU for animal studies, were followed for handling animals [44]. The Research Ethics Committee of the Faculty of Pharmacy, Future University in Egypt, Cairo, Egypt approved the study protocol. The study was approved under an approval number: REC-FPFUE-03/2024.

#### 2.8.2. Groups and Induction

To start, before exposure, the mice’s back hair was shaved using shaving cream, and the shaved patch was exposed to UVA (365 nm) light for 5 days/week for 4 weeks. The distance between the mice and the lamp (EL Series UV Lamps; Analytik Jena US LLC., Upland, CA, USA) was 15 cm [45]. After wrinkles were induced, treatment was initiated for 14 consecutive days. 

The mice were grouped as follows. First group (healthy control mice (NC)): these mice had not been exposed to UVA radiation and were used as a control for normal skin. Second group (positive control group, exposed untreated mice (PC)): these mice developed wrinkles after exposure to the UVA lamp but received no treatment; these served as the baseline for comparison. Third group (wrinkled mice treated with free nanofiber patch without RA (free-P): these mice developed wrinkles following exposure to the UVA lamp and were exposed to free-P that was applied daily for the full treatment course. Fourth Group: (wrinkled mice treated with RA solution (liquid RA): These mice developed wrinkles and received treatment with a liquid RA solution (without being in nano form). This patch contained the active ingredient (RA stem cell extract) and 1 mL was sprayed onto wrinkled skin (1 mg/mL) once daily for 14 days. Finally, the fifth group-wrinkled mice treated with the nanofiber patch (medicated-P): these mice developed wrinkles using a UVA lamp and were treated with a nanofiber patch containing stem cell extract with RA at a concentration of 1 mg/mL (1 cm × 1 cm patch). The patch was applied directly to wrinkled skin and replaced with a new patch daily for 14 days.

Prior to treatment, the dorsal region of the mice was shaved using a depilatory cream, and the exposed area was subjected to UVA radiation (365 nm) for 5 days per week over 4 weeks. The distance between the mice and the UVA lamp (EL Series UV Lamps; Analytik Jena US LLC., Upland, CA, USA) was maintained at 15 cm. After the induction of wrinkles, treatment was initiated and continued for 14 consecutive days.

#### 2.8.3. Tissue Extract

After administering a lethal dose of thiopental (IP 200 mg/kg) to end the treatment period, a 2 × 2 cm skin patch was excised from each mouse, and the tissue was divided into two parts. One part was homogenized in 10% phosphate-buffered saline (PBS) and stored in aliquots to estimate the corresponding biomarkers. The remaining samples were stored in 10% formaldehyde for histological analysis.

### 2.9. In Vivo Characterization of Extracted Rosmarinic Acid Electrospun Nanofiber Patch

#### 2.9.1. Skin Histopathology

Three mice from each group had their dorsal skin excised and removed, fixed for a day in 10% formaldehyde, and cleaned with tap water before being dehydrated using dilutions of alcohol. The specimens were washed with xylene and then immersed in paraffin for a day at 56 °C in a hot air oven. A sledge microtome was used to segment paraffin blocks at a thickness of 4 μm. Tissue sections were then collected on glass slides, deparaffinized, and stained with hematoxylin and eosin (H&E) to prepare them for inspection using a light electric microscope (Evident (Olympus Scientific Solutions), Tokyo, Japan) [46].

#### 2.9.2. The Assessment of the Effect of the Medicated Nanofiber Patch on the Nrf2/Keap1 Signaling Pathway

Skin homogenates were prepared as described by the manufacturer, and the cellular content of the chosen parameters was assessed using the corresponding ELISA kit (Nrf2, Keap1; My BioSource, San Diego, CA, USA Catalogue #: MBS2516218 and MBS2890837, respectively).

#### 2.9.3. The Assessment of the Effect of the Medicated Nanofiber Patch on the Antioxidant Defenses in the Skin

The content of reduced glutathione (GSH), glutathione peroxidase (GPx), superoxide dismutase (SOD), and malondialdehyde (MDA) were assessed in the skin according to previously described methods [47]. (GSH, GPx, SOD, and MDA; Biodiagnostic, Giza, Egypt; catalog #s: GP-2523, GP 2524, SD 2521, MD-2529, respectively).

#### 2.9.4. Statistical Analysis

The results were presented as the mean ± SD. The analysis of variance (ANOVA) was used to determine whether there were any significant group differences, and Tukey’s post hoc test was performed. In addition to these, an unpaired *t*-test was used to assess differences between any two groups when needed. The statistical significance threshold was set at *p* < 0.05 using version 10.2.2. GraphPad Prism^®^ software package (GraphPad Software, San Diego, CA, USA).

## 3. Results and Discussion

### 3.1. Callus Initiation

As a matter of fact, establishing an efficient callus induction protocol is critical for producing bioactive compounds in plant cell cultures [48]. In this study, the callus was successfully initiated from *SM* shoot tip explants, which are small parts that were cut off the plant’s shoot tip.

### 3.2. Eliciation Process with Jasmonic Acid (JA) and Salicylic Acid (SA)

Indeed, the choice of the elicitor is crucial for enhancing the yield of secondary metabolites. In this study, the eliciation treatments used two signaling molecules, JA and SA. These compounds are known to stimulate defense responses and secondary metabolite production in many plant species. In this experiment, JA augmented RA production by 200% while SA increased RA production by 81%. These results were in line with the following study that confirms that exposing SM cell cultures to these elicitors can enhance RA production [49].

**Figure 1:**
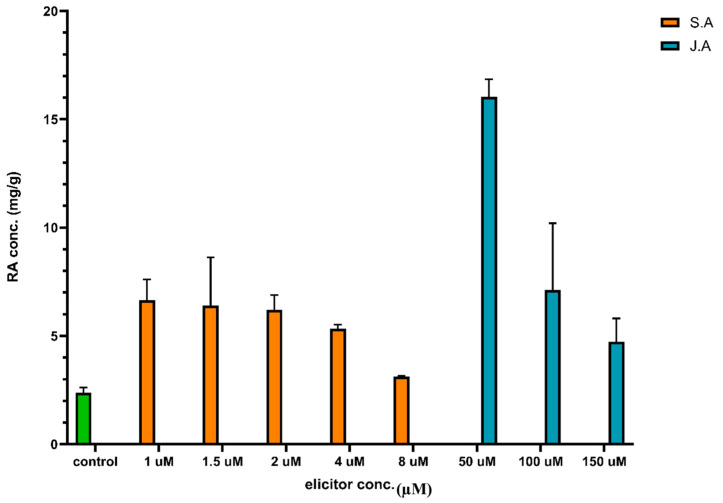
A chart showing the effect of different concentrations of Jasmonic acid and Salicylic acid on Rosmarinic acid (RA) yield in the *Salvia miltiorrhiza* stem cell extract compared with the (0) control: RA yield without elicitors, which is indicated with the green column.

The effects of different concentrations of JA and SA on the yield of RA from callus extracts were evaluated. As shown in Figure 1, it was observed that the lowest concentration of JA (50 μM) resulted in the highest RA content of 16 mg/g dry weight (DW) 

In the case of SA, the same pattern was noticed, where the lowest concentration of 1 μM gave a maximum RA yield of 6.6 mg/g DW (Figure 1). Likewise, it was noted that using higher SA concentrations (1.5, 2, 4, and 8 μM) was less effective at enhancing RA production.

When the optimal concentrations of the two elicitors were compared, 50 μM JA treatment led to a substantially higher RA yield (16 mg/g DW) compared to 1 μM SA (6.6 mg/g DW). This indicates that JA is a more potent elicitor of RA biosynthesis than SA in SM cell cultures. This finding aligns with the broader pattern noted in various medicinal plant studies [49,50]. This phenomenon can be attributed to the previous explanation that moderate doses of elicitors enhance the production of bioactive compounds and higher doses may be less effective or even inhibitory [50,51]. 

In fact, the mechanisms underlying these concentration-dependent effects involve complex interactions within the plant’s physiological and metabolic pathways. At optimal concentrations, JA and SA can activate specific signal transduction pathways that enhance the synthesis of secondary metabolites, which are often involved in plant defense mechanisms [51,52,53]. However, at higher concentrations, these compounds might trigger a stress response that could be detrimental to the overall plant growth and metabolism, thereby reducing the yield of target metabolites [53,54,55,56].

In summary, the concentration-dependent effects of JA and SA on secondary metabolite production in medicinal plants focus on the importance of tailored biotechnological practices. Thus, by optimizing the elicitor concentrations, it is possible to significantly enhance the yield of bioactive compounds, which is crucial for pharmaceutical development and other tissue culture applications.

### 3.3. Design Optimization for Eliciation Treatment

**Figure 2:**
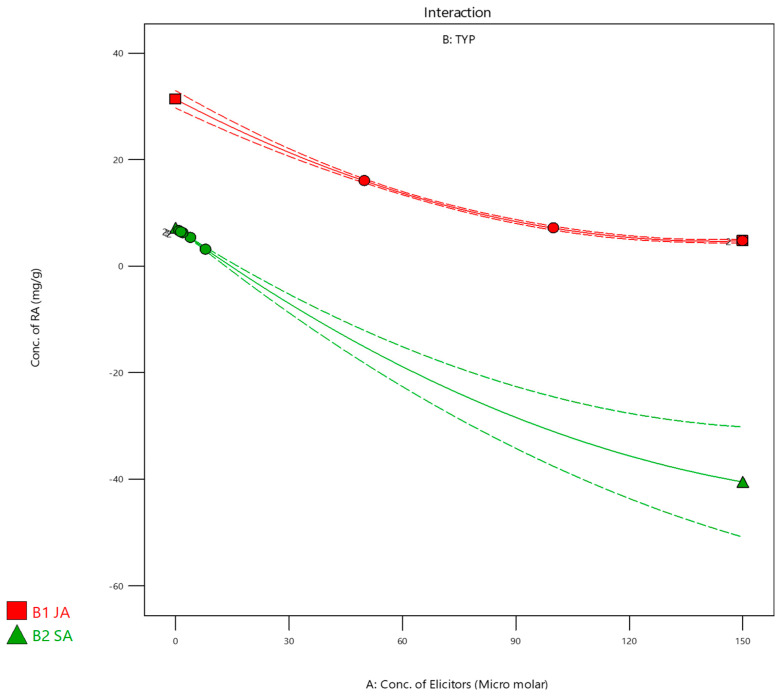
The effect of Jasmonic acid and Salicylic acid elicitor concentrations on Rosmarinic acid level, solid lines represent the predicted responses from the statistical model and dashed lines Indicate observed experimental responses.

**Figure 3:**
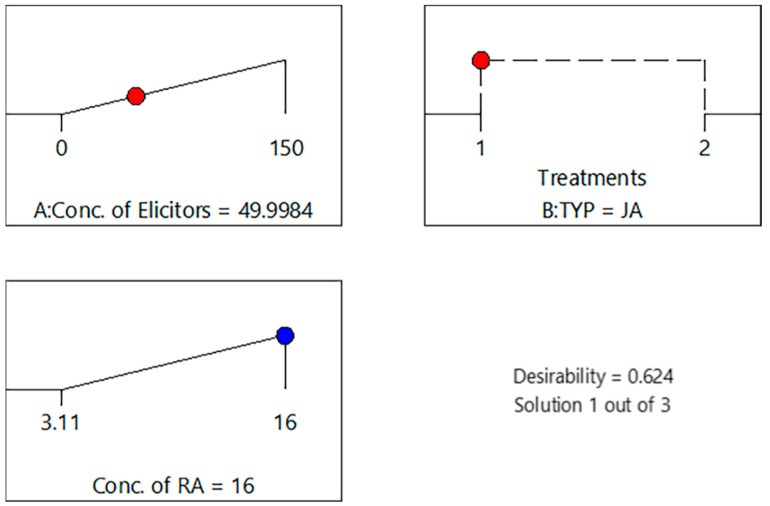
Desirability ramps for the ideal elicitor concentration where red dots represent jasmonic acid concentration and blue dots represent rosmarinic acid concentration.

Treatments were applied with different concentrations and types of elicitors, which were the independent variables to study their effect on the dependent variable, the RA level, as shown in Table 2. There were no issues with the data or model, as indicated by the statistical acceptance of the predicted and adjusted R^2^ values [57], as shown in Table 3 and Figure 2 of the desirability report created by Design Expert software, demonstrating that JA 50 µM (Desirability = 0.624) was the ideal elicitor concentration selected, as shown in Figure 1, confirming that JA was more effective than that of SA to obtain higher total phenolic yields in callus cultures of SM [56]. Figure 3 shows the interaction of both JA and SA elicitors and shows the best concentration for elicitor treatment, which was 50 µM, resulting in 16 mg/g RA. 

### 3.4. In Vitro Characterization of Extracted Rosmarinic Acid Electrospun Nanofiber Patch

#### 3.4.1. Drug Content

The value of theoretical RA content compared to the actual RA content was 85.8% ± 2.81, which did not show any significant difference using the *t*-test *p* value (*p* > 0.05) and met the United States Pharmacopoeia (USP 39-NF 34) drug content standards [58].

#### 3.4.2. In Vitro Release of Extracted Rosmarinic Acid Electrospun Nanofiber Patch

**Figure 4:**
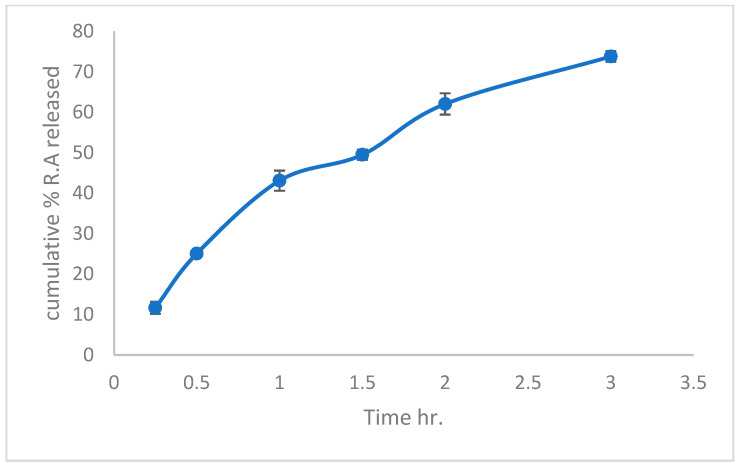
In vitro release profile of Rosmarinic acid from nanofiber patch in acetate buffer pH 5.5. Data are presented as mean+/− SD at *p* < 0.05 *n* = 3.

The RA release profile from the PU nanofiber patch is shown in Figure 4. Owing to the release of RA at approximately 11% and 25%, the nanofibers exhibited a burst release after the first 30 min. Within three hours, the remaining 66–77% of the medication in the nanofibers was released at somewhat slower rates. Other researchers have also reported this [59]. The first stage of drug release from the nanofiber patches was probably caused by drug molecules that were dissolved on the surface of the fibers and quickly dissolved in a buffer solution for 30 min. In contrast, the second stage of drug release from the fiber centers occurs gradually and continuously [60,61].

#### 3.4.3. Swelling Degree of Extracted Rosmarinic Acid Electrospun Nanofiber Patch

When treating wrinkles, the ability of patches to swell is a crucial characteristic. The swelling capacity of the PU/RA nanofiber patches was evaluated using an acetate buffer with a pH of 5.5. The high acetate buffer absorption value of the PU/RA nanofiber (311% ± 0.57) after 24 h is attributed to its hydrophobic character. The experiment was carried out in triplicate and the numbers mentioned above represent percentages +/− standard deviations. Combining the hydrophilic (RA) and hydrophobic (PU) components, because of the hybrid nature of the PU/RA core–shell scaffold, the surfaces have a hydrophilic quality that increases the percentage of acetate buffer absorbed [62].

#### 3.4.4. Mechanical Strength of the Rosmarinic Acid-Loaded Nanofiber Patch

The prepared nanofiber patch exhibits a tensile strength of 2.6 ± 0.3 MPa and demonstrates moderate mechanical performance, which is suitable for wrinkle treatment applications. PU is known for its flexibility and durability, while its nanofibrous structure provides enhanced surface area and porosity, potentially improving skin interaction and comfort. Although a tensile strength of 2.6 ± 0.3 MPa is sufficient for typical use. Given the importance of comfort and flexibility in wrinkle treatments, this strength appears adequate [63,64].

#### 3.4.5. Fourier Transform Infrared Spectroscopy (FTIR) Analysis

**Figure 5:**
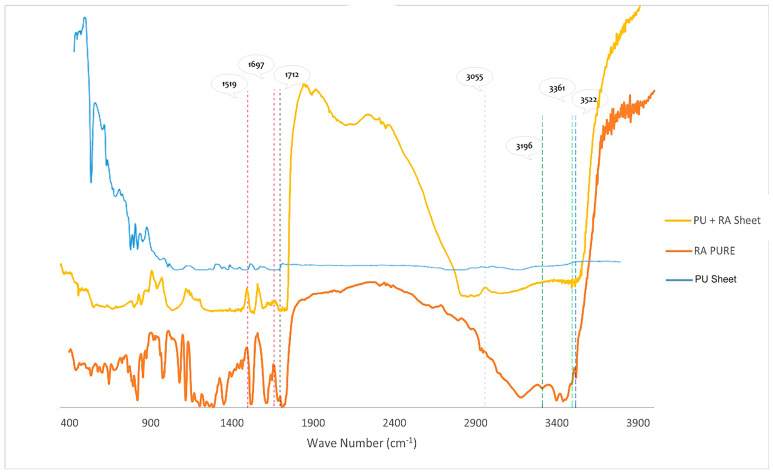
FTIR spectrum of Polyurethane/Rosmarinic acid electrospun nanofiber.

In the FTIR spectrum of PU/RA, as shown in Figure 5, there was no change in the characteristic peaks of RA, there was no new compound resulting from the mixture of PU and RA, and the stretching vibration of phenolic O-H was responsible for the absorption bands at 3361 cm^−1^ and 3522 cm^−1^. O-H acid was associated with a peak at 3196 cm^−1^. The peak at 2937 cm^−1^ is ascribed to C-H stretching, whereas the peak at 3055 cm^−1^ is related to the frequency of =C-H aromatic stretching. The C=O stretching vibration was responsible for the bands at 1712 cm^−1^ and 1697 cm^−1^. The bending vibration of C-H was responsible for the bands ranging from 864 cm^−1^ to 634 cm^−1^ [37].

#### 3.4.6. Impact of Storage on the Prepared Patch on Drug Content and In Vitro Release

The results of stability studies 3 months after preparation of the PU/RA electrospun nanofiber patch showed no difference in the drug loading content percentage of RA from the first prepared patch with 85.8% ± 2.81 and no significant difference using the *t*-test (*p* > 0.05). [65].

**Figure 6:**
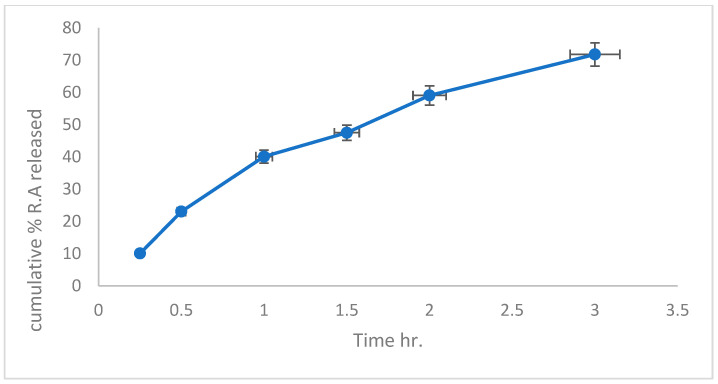
In vitro release profile of Rosmarinic acid electrospun nanofiber in acetate buffer pH 5.5 showing the patch’s stability after 3 months of preparation. Data are presented as mean+/− SD at *p* < 0.05 *n* = 3.

The results of stability studies after 3 months from patch preparation showed no significant difference in the dissolution release using the *t*-test (*p* > 0.05). The release of RA at 10% and 23% caused the nanofibers to show an initial burst release at the end of the first 30 min. The remainder of the nanofibers’ medication was released within three hours at a significantly slower rate, as shown in Figure 6 [66].

### 3.5. Scanning Electron Microscope and Surface Roughness

**Figure 7:**
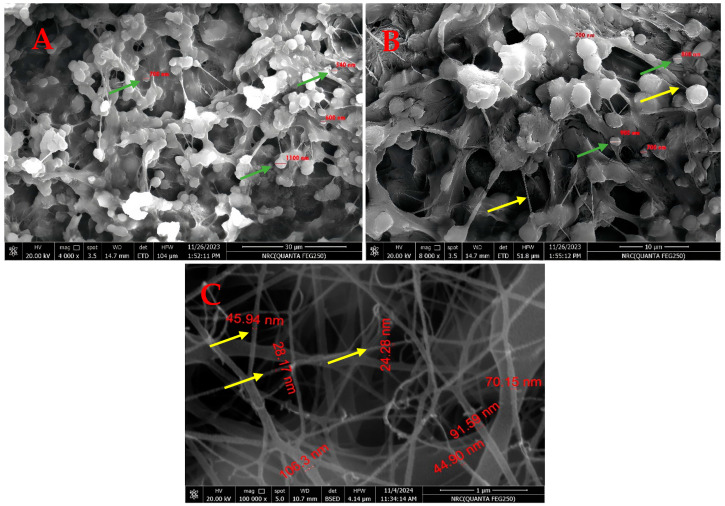
SEM images of electrospun RA/PU nanofiber patch, polymer solution 10%wt with flow rate 0.8 mL/h and 25 kV, (**A**) 30 µM, (**B**) 10 µM, (**C**) Free Polyurethane. Green arrows sign to the nanospheres of extracted rosmarinic acid, yellow arrows sign to the uniformly loaded nanofibers.

Nanospheres of extracted RA shown with green arrows were uniformly loaded on the nanofiber surface with yellow arrows, increasing their surface roughness, as shown in Figure 7A,B. Also, nanofibers of Polyurethane polymer were uniformly formed as shown in Figure 7C. The SEM images in Figure 7 show nanospheres uniformly loaded onto the nanofiber surface. Coaxial electrospinning technology covers nanofibers with nanospheres, thereby increasing their surface roughness. As a result, nanofibers and nanospheres could be injected together to form an electrospun nanofiber patch at the intersection of the two pumped syringes. Finally, the nanofiber patch was collected. The mean fiber diameter is 69.24 ± 13.16, and the mean particle diameter is 613.3 ± 43 (reading = average value ± Standard Error (SE)) [67].

**Figure 8:**
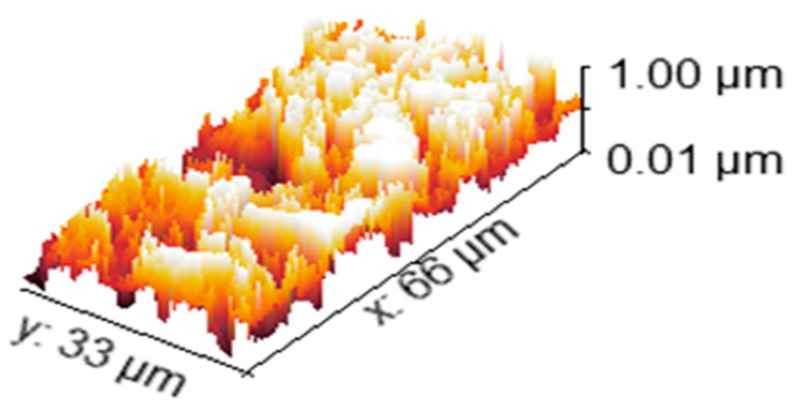
Surface roughness images for Rosmarinic acid/Polyurethane nanofiber patch.

The surface roughness of the RA nanofiber is shown in Figure 8, which illustrates how a rough surface is obtained and how it differs slightly from the PU surface. The surface roughness of both nanoparticles, as determined by roughness evaluation metrics including Ra, Rt, Rv, and Rp, is summarized in Table 4. The surface roughness was determined by translating a 2D SEM image into a 3D image. Ra considers the notches and heights. Rv refers to the gap depth, and Rp indicates only the height. The roughness of the surface is shown by both the height and cracks, which may promote adhesion to the host tissue. The high heights (Rt values) compared to their analog for notches (Rp values) suggest that physical interaction is the primary mode of connection with the surroundings. In other words, heights act as hooks to connect with nearby molecules. When RA and PU were combined, the nanofiber surface became rougher, promoting physical adhesion to the skin surface [42].

#### 3.5.1. Physical Appearance and Skin Histopathology

**Figure 9:**
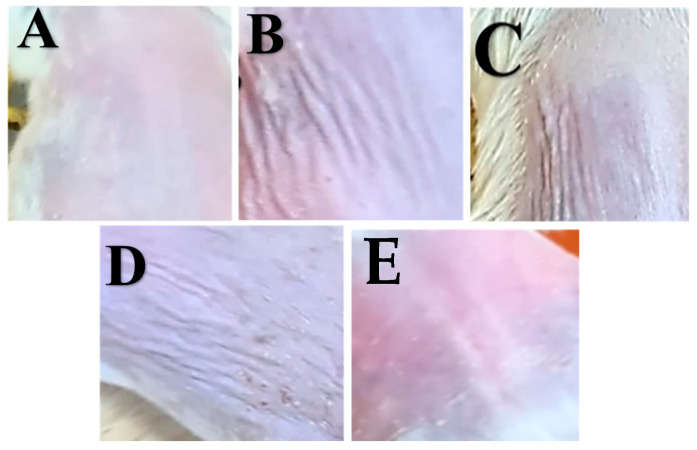
Photograph of a sample of the mice’s back after treatment. (**A**) Normal control (NC) group, (**B**) positive control (PC) group, (**C**) free patch group (Free-P), (**D**) liquid RA group, (**E**) medicated nanofiber patch-treated group (Mediated P).

**Figure 10:**
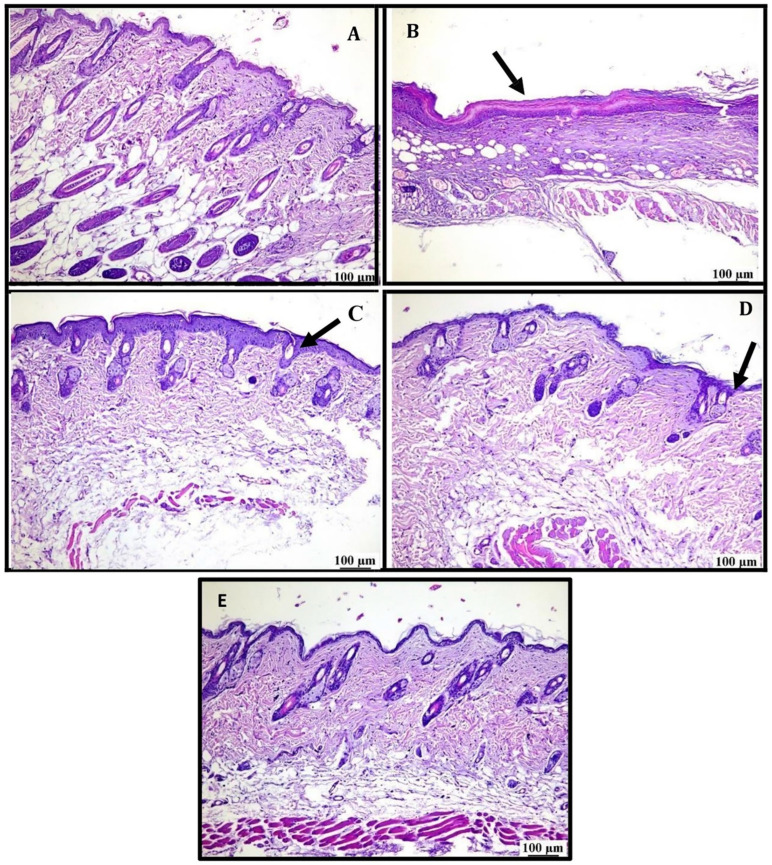
Skin histopathological images. (**A**) Normal control (NC) group, (**B**) positive control (PC) group, (**C**) free patch group (Free-P), (**D**) liquid RA group, (**E**) medicated nanofiber patch-treated group (Medicated P). Arrows represent the variation in epidermal thickening and the presence of necrotic tissues.

The PC group skin exhibited visible signs of photoaging, including fine lines and deeper wrinkles across the surface and rough skin (Figure 9B) compared to normal skin (Figure 9A). However, in the medicated nanofiber-treated group, the skin displayed a significant reduction in these aging signs, with skin texture improvement (Figure 10E). On the other hand, wrinkles were still visible in all other groups (Figure 10B–D), including those treated with the liquid patch, which showed apparent dryness of the skin (Figure 10D). 

A microscopic examination of the normal control (NC) group revealed a normal histological structure of the skin, including the epidermis and dermis (Figure 9A). The positive control (PC) group showed marked epidermal thickening with a thick necrotic crust covering the epidermis. Some of the examined sections showed epidermal ulceration, whereas others exhibited dermal scarring (Figure 9B). Mice treated with the patch free of medication (Free-P) showed minor improvement, as the examined sections revealed epidermal thickening (Figure 9C). Mild improvement was detected upon treatment with liquid RA extract, as the examined sections showed focal dermal scarring and mild destruction of collagen (Figure 9D). The histopathological analysis conducted on the skin samples across different treatment groups reveals varied levels of epidermal and dermal integrity. The normal control (NC) group demonstrated typical histological structures, showcasing a healthy epidermis and dermis without any observable abnormalities [68]. In addition to that. The positive control (PC) group, however, exhibited significant pathological alterations. The most prominent findings included pronounced epidermal thickening and the presence of necrotic tissues. These changes indicate severe skin damage and compromised structural integrity, which could be attributed to exposure to harmful agents or simply the sun’s ultraviolet radiation [69]. Upon treatment with the free patch, there was a little improvement in skin condition and that can be explained by the concept of hydration [70].

The liquid-treated group demonstrated a mild level of recovery with evidence of focal dermal scarring and mild collagen destruction. These findings point to an intermediate level of tissue repair and remodeling, indicating that the treatment in this group had some efficacy but was insufficient for full restoration of the skin’s normal architecture.

The most apparent recovery was observed in the medicated group, where the skin appeared to have returned to an almost normal state. Both general and higher magnification images depicted intact and healthy epidermal and dermal structures. This suggests that the medication applied had a significant therapeutic effect, effectively promoting healing and restoring skin integrity without residual damage. 

These results collectively highlight the varying degrees of effectiveness in the treatments applied, with the medicated group showing the most promising outcome in terms of histological restoration. The comparison between the PC group and other treatment groups emphasizes the importance of targeted therapeutic strategies in managing skin damage and promoting recovery. Further investigation into the mechanisms driving these differential outcomes could provide valuable insights into enhancing skin repair strategies. 

Finally, the medicated patch (Medicated-P) was close to normal skin (Figure 9E) after the induction of wrinkles using UVA radiation.

#### 3.5.2. The Medicated Nanofiber Patch Targets the Nrf2/Keap1 Signaling Pathway

**Figure 11:**
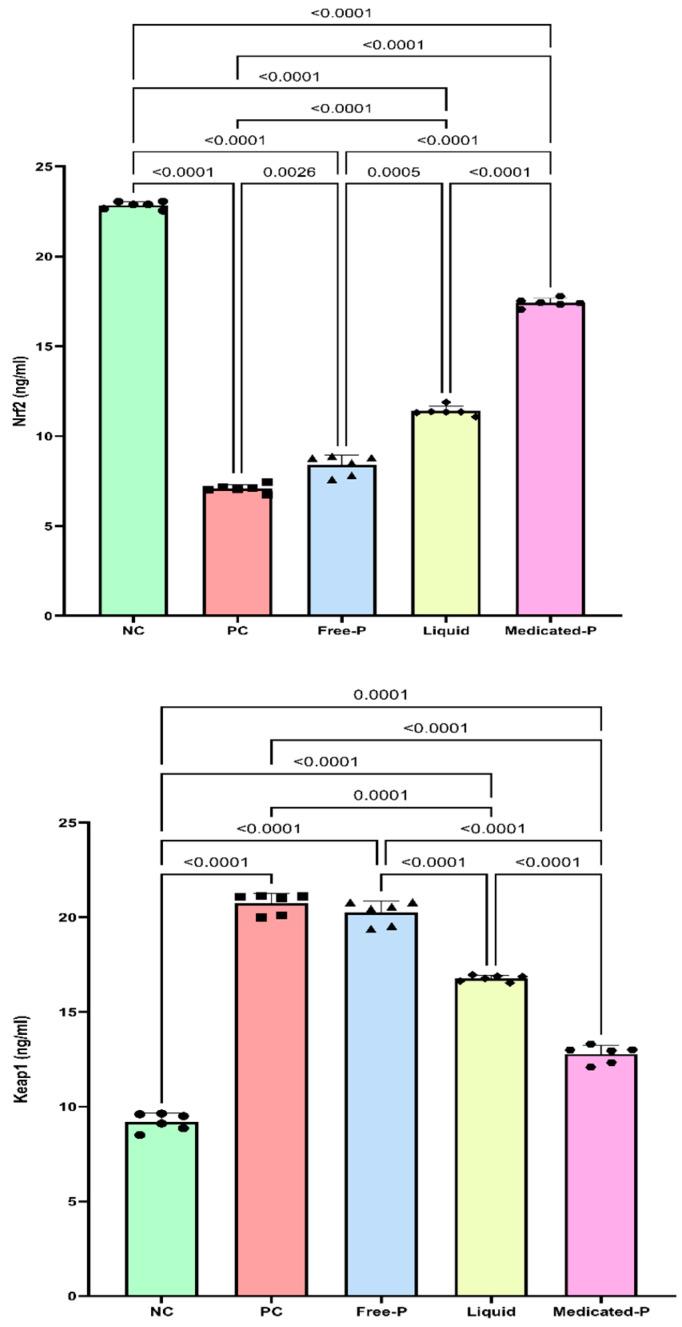
Effect of different treatments on skin Nrf2 and Keap1 content. Data are presented as mean ± SD (*n* = 6). Statistical analysis was carried out using one-way ANOVA followed by Tukey’s Multiple Comparison test, and the used *p* value is shown for each comparison in the graph. Each shape over the bar-chart represents a sample that was tested.

Ultraviolet radiation greatly increases the oxidative burden on the skin tissues. This effect was demonstrated by a significant decrease in the tissue content of the transcription factor Nrf2 and a great increase in the tissue content of Keap-1 by 69% and 1.2-fold, respectively, compared with that in the NC group. However, the medicated nanofiber patch greatly augmented the transcription factor Nrf2 by 1.4-fold and mitigated the increase in Keap1 by 38.3% compared with the positive control. The choice of targeting the KEAP1/NRF2 pathway in exploring the effect of *Salvia miltiorrhiza* extract for treating wrinkles is based on the crucial role this pathway plays in cellular protection and skin health. The KEAP1/NRF2 pathway regulates the body’s response to oxidative stress by activating NRF2, which in turn induces the expression of antioxidant and protective genes. Oxidative stress is a significant contributor to skin aging and the formation of wrinkles due to the breakdown of collagen and other structural components of the skin. By modulating this pathway, *Salvia miltiorrhiza* extract can enhance the skin’s defense mechanisms, boost collagen production, and reduce oxidative damage, ultimately leading to improved skin elasticity and a reduction in wrinkles [71]. Although the medicated liquid sprayed on the skin of mice showed a slight increase in Nrf2 levels and a slight decrease in keap-1 levels, the patch showed a significant difference, with a 1-fold higher Nrf2 level and 24% lower keap1 than the group treated with the liquid patch; this can be owed to the hydrating effect of the nanofibers as they have hygroscopic characteristic that allows them to retain moisture [72]. Notably, the free unmedicated patch showed a small yet significant change in Nrf2 levels in the tissues by only 18%, which can be owed to the capabilities of the PU polymer in self-healing, its low inflammatory potential, and biocompatibility with healthy skin cells [23]. Conversely, no significant change in Keap1 levels was observed upon the application of the unmedicated carrier-only patch compared to the untreated positive control group, as shown in Figure 11. Ultraviolet radiation significantly downregulated Nrf2 levels, indicating oxidative stress-induced disruption of cellular antioxidant defense mechanisms [73]. This indicates a potential mechanism through which the patch enhances Nrf2-mediated antioxidant responses and ensures that its application is beneficial as an anti-wrinkle formulation. Another study suggested that activating the antioxidant myriad through Nrf2 activation has a photoprotective effect on the skin [74].

#### 3.5.3. Medicated Patch Had a Positive Impact on Cellular Oxidative Stress: Modulation of Glutathione, Glutathione Peroxidase, Superoxide Dismutase, and Malondialdehyde Levels

**Figure 12:**
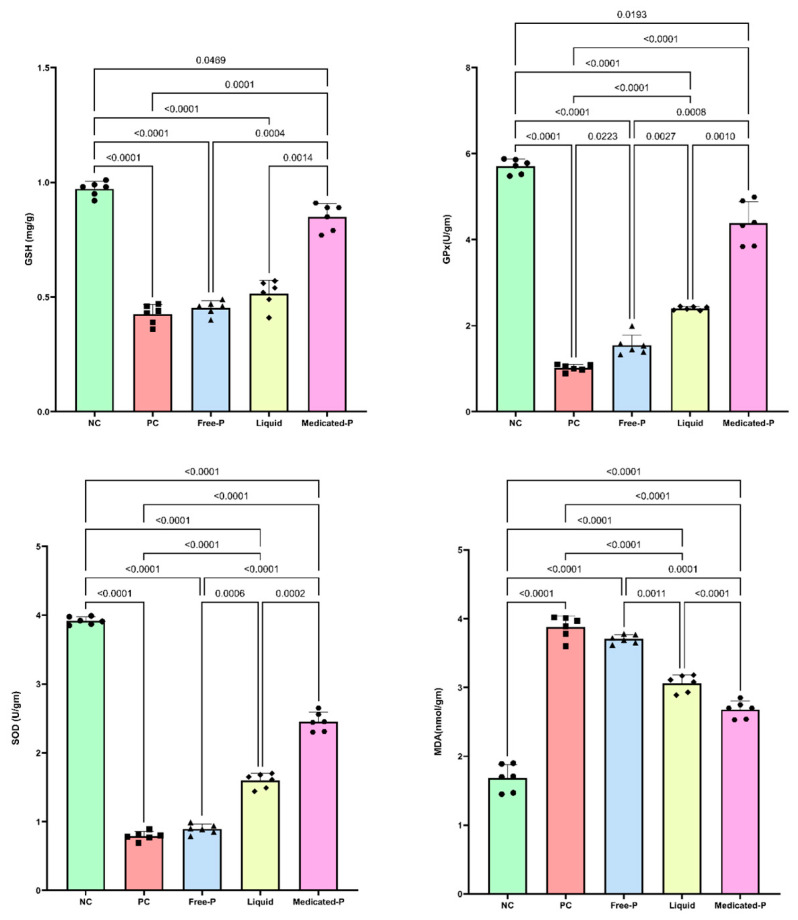
Effect of different treatments on skin GSH and GPx, SOD, MDA. Data are presented as mean ± SD (*n* = 6). Statistical analysis was carried out using one-way ANOVA followed by Tukey’s Multiple Comparison test, and the used *p* value is shown for each comparison in the graph. Each shape over the bar-chart represents a sample that was tested.

The outcomes of this study demonstrated a balance in the cellular oxidative stress levels, evident by a significant incline in GSH, GPx, and SOD levels following the application of the medicated patch compared to the diseased positive control by 1-fold, 3.3-fold, and 2-fold, respectively. By the same token, the group treated with the sprayed liquid patch showed an amplification in the GSH, GPx, and SOD levels by 21.5%, 51%, and 99.7%, respectively, as shown in Figure 12. In addition, the free, unmedicated patch showed negligible changes in tissue antioxidant parameter levels. One of the key findings of this study was the significant elevation in antioxidant parameters levels because the exposure of the skin to UV greatly increases reactive oxygen species due to the oxidation products that accumulate in the skin, which causes skin aging that needs to be combatted [73]. Thus, the observed increase in GSH levels underscores the ability of the medicated patch to bolster the antioxidant capacity, highlighting its potential as a therapeutic intervention in conditions characterized by an oxidative imbalance [75]. Moreover, the augmentation of GPx activity following treatment with the medicated nanofiber patch further supports its antioxidant properties. The role of GPx against ROS was highlighted in a study showing that the increase in GPx activity suggests enhanced antioxidant defense mechanisms, which could contribute to cellular protection against oxidative stress-induced damage and the patch’s anti-wrinkle effect [76]. Moreover, the efficacy of the nanofiber patch can be explained by its advanced controlled release profiles, responding to environmental stimuli or internal factors; this capability allows for prolonged and more pronounced therapeutic effects, crucial for maintaining elevated antioxidant levels in skin tissues over time [77,78].

#### 3.5.4. The Medicated Patch Subsides Skin Lipid Peroxidation

Following UV exposure, the results revealed a profound increase in the lipid peroxidation marker MDA levels by 1.4-fold compared to the normal group. However, treatment with the medicated patch significantly decreased MDA levels by 31.5% compared to the untreated control, as shown in Figure 12, underscoring its efficacy in attenuating UV-induced lipid peroxidation. Interestingly, the medicated patch-treated group showed a 13% decrease in tissue MDA compared with the liquid patch. In contrast, the application of the free patch yielded no significant change in MDA levels, suggesting that the observed reduction in MDA was specific to the formulation of the medicated patch, rather than mere occlusive effects. This study demonstrated a profound attenuation of the UV-induced lipid peroxidation marker MDA following treatment with the medicated patch and an increase in the protective metalloenzyme SOD. This observation highlights the targeted efficacy of the medicated patch in mitigating UV-induced oxidative damage and presents a promising approach for dermatological applications. The results of this study are in line with another study in which UV-induced skin damage was facilitated by an increase in SOD activity and a decrease in MDA content [76,79].

## 4. Conclusions

In conclusion, RA nanofiber patches extracted from SM stem cells have the potential to initiate a new trend in skincare and delivery systems within pharmaceutical biotechnology and cosmeceuticals. This approach contributes to the development of a sustainable and eco-friendly dosage form. Moreover, this study underscores the anti-wrinkle effects of SM stem cell extract, which operates intracellularly via the Nrf2/Keap1 pathway, along with its significant antioxidant properties. Collectively, the formulation represents a valuable advancement in creating a clinically applicable anti-wrinkle and antioxidant product based on plant stem cell extracts that is safe and eco-friendly. This development will greatly benefit individuals seeking safe, sustainable, and effective anti-aging solutions. Future studies are recommended to further explore and confirm the therapeutic potential of this formulation. Also, the effect of nanofibers on the opposite gender will be examined in a subsequent study.

## Figures and Tables

**Table 1:** D-optimal design (DOD) of RA level in *SM* callus culture.

Factors	Factor Type	Levels	
		Low	High
X1: Conc. of elicitors X2: Type of elicitors	Numeric Categoric	1 µM SA	150 µM JA
Responses		Desirability constraints	
Y1: Concentration of RA		Maximize	

RA = Rosmarinic acid, SM = *Salvia miltiorrhiza*, X1 = Conc. of elicitors, X2 = Type of elicitors, Y1 = Concentration of RA.

**Table 2:** Experimental runs, independent variables, and measured response of the D-Optimal experimental design of Rosmarinic acid yield improvement.

Treatments	Conc. of Elicitors (X1) (µM)	Type of Elicitors (X2)	Conc. of RA (mg/g)
T1	50	JA	16 ± 0.29
T2	1	SA	6.65 ± 1.63
T3	1.5	SA	6.4 ± 0.16
T4	2	SA	6.21 ± 1.17
T5	4	SA	5.32 ± 0.33
T6	100	JA	7.13 ± 0.38
T7	150	JA	4.74 ± 0.39
T8	8	SA	3.114 ± 0.066

**Table 3:** Output data of the elicitors analysis and predicted and observed values for the selected elicitor.

Source	RA Conc. (mg/g) (Y_1_)
*p* value	<0.0001
Model	Quadratic
X_1_ = A = Conc. of Elicitors	0.0002
X_2_ = B = Type of Elicitors	0.0002
Adequate precision R^2^	138.1224 0.9996
Adjusted R^2^	0.9991
Predicted R^2^	−219.7502
Significant factors	X_1_, X_2_
Predicted value of the selected patch	49.99
Observed value of the selected patch	50 ± 0.529
The regression equation of the fitted model	+31.33 − 0.3.47 × A + 0.0012 × A^2^

**Table 4:** Roughness parameters of RA and PU nanocomposites, polymer solution 10%wt with flow rate of 0.8 mL/h and 25 kV.

Composition	Ra (nm)	Rt (nm)	Rv (nm)	Rp (nm)
10% PU polymer + Rosmarinic acid	62.7	726.0	325.7	400.2

(Ra): the notches and heights; (Rt values): the high heights, (Rv): the gap depth; (Rp): the height; (PU): Polyurethane.

## Data Availability

The data are available upon request.
